# Impact of atrial fibrillation ablation on long‐term outcomes in patients with tachycardia‐bradycardia syndrome

**DOI:** 10.1002/joa3.12738

**Published:** 2022-05-19

**Authors:** Shohei Kataoka, Koichiro Ejima, Kyoichiro Yazaki, Miwa Kanai, Daigo Yagishita, Morio Shoda, Nobuhisa Hagiwara

**Affiliations:** ^1^ Department of Cardiology Tokyo Women's Medical University Shinjuku‐ku Japan; ^2^ Clinical Research Division for Heart Rhythm Management, Department of Cardiology Tokyo Women's Medical University Shinjuku‐ku Japan

**Keywords:** atrial fibrillation, permanent pacemaker implantation, pulmonary vein isolation, tachycardia‐bradycardia syndrome

## Abstract

**Background:**

Reports of long‐term outcomes after atrial fibrillation (AF) ablation for tachycardia‐bradycardia syndrome (TBS) are limited. This study aimed to investigate the impact of radiofrequency catheter ablation (RFCA) on clinical outcomes in patients with TBS.

**Methods:**

Among 1669 patients who underwent AF ablation between January 2010 and April 2020, we retrospectively enrolled 53 patients (62.3% males; age, 67.1 ± 7.0 years) who had been diagnosed with TBS before RFCA for paroxysmal AF (TBS group). After 1:2 propensity score‐matching based on age, gender, AF type, and left atrial dimension, 106 patients were assigned to the control group (non‐TBS group). The atrial tachyarrhythmia (ATA) recurrence rate and rate of avoidance of permanent pacemaker implantation (PMI) were examined.

**Results:**

During a median follow‐up period of 37.7 months, the ATA recurrence rate after a single ablation procedure was significantly higher in the TBS group than in the non‐TBS group (51.0% vs. 38.5%; log‐rank *p* = .008); however, the ATA recurrence rate after the final ablation procedure did not significantly differ between groups. In the TBS group, the rate of PMI avoidance after AF ablation was 92.5%. A Cox‐regression multivariate analysis revealed that the presence of non‐pulmonary vein/superior vena cava premature atrial contractions (odds ratio, 3.38; 95% confidence interval, 1.49–7.66; *p* = .004) was an independent predictor of ATA recurrence in the TBS group.

**Conclusions:**

Patients with TBS had higher ATA recurrence rates after the first ablation procedure compared to those without TBS. However, ATA recurrence after AF ablation did not necessarily result in PMI for TBS patients.

## 1 INTRODUCTION

Tachycardia‐bradycardia syndrome (TBS) is characterized by atrial tachyarrhythmia (ATA), including atrial fibrillation (AF), atrial flutter, or atrial tachycardia, followed by prolonged sinus pauses, resulting in Adams‐Stokes syndrome. Historically, permanent pacemaker implantation (PMI) has been performed for patients with TBS and AF.^1^ However, radiofrequency catheter ablation (RFCA) has been recently reported as an alternative therapy for TBS. Previous studies have shown better outcomes of RFCA for TBS regarding the rate of sinus rhythm maintenance compared with PMI plus anti‐arrhythmic drugs (AADs).^2^, ^3^ However, it remains controversial whether RFCA is feasible as first‐line therapy for TBS. Additionally, the clinical outcomes in TBS patients with ATA recurrence after AF ablation have not been fully evaluated. This study aimed to investigate long‐term clinical outcomes in patients who had undergone AF ablation for TBS.

## 2 METHODS

### 2.1 Inclusion and exclusion criteria

This study was a retrospective analysis of the AF database of Tokyo Women's Medical University. Between January 2010 and April 2020, a total of 1669 patients underwent AF ablation at our institution. We excluded 32 patients who underwent AF ablation using balloon technology and 67 patients who underwent cardiac implantable electronic devices implantation prior to AF ablation. Of the 1570 remaining patients, we retrospectively enrolled 53 patients who had been diagnosed with TBS and paroxysmal AF prior to RFCA (TBS group). TBS was diagnosed when sinus pause of more than 3 s after the termination of AF was detected using electrocardiography (ECG) or Holter ECG, which resulted in bradycardia‐related symptoms, such as dizziness or syncope.^4^ After 1:2 propensity score‐matching based on age, gender, AF type, and left atrial dimension, 106 patients were selected as the control group (non‐TBS group).

### 2.2 Data collection and ethical consideration

Well‐trained physicians performed data collection based on individual chart reviews. Age, gender, body mass index, AF type, comorbidities, regular medications, echocardiographic parameters, and laboratory data were used as baseline characteristics. Procedural characteristics were also assessed. The maximum P‐wave duration (PWD) determined by the surface 12‐lead ECG was measured as an indicator of atrial remodeling. The maximum PWD was defined as the interval from the earliest onset to the latest offset of the 12‐lead ECG before AF ablation. The study protocol conformed to the ethical guidelines of the 1975 Declaration of Helsinki and was approved by the Institutional Review Board and Ethics Committee of the Tokyo Women's Medical University. Written informed consent was obtained from all patients.

### 2.3 Ablation procedure

Details of the AF ablation procedure performed at our institution have been previously described.^5^ Before the procedure, all AADs were discontinued for at least five half‐lives. A decapolar catheter was inserted from the right femoral vein and placed in the coronary sinus beyond the three o'clock location of the mitral annulus. We measured the interatrial conduction time (IACT), defined as the interval from the earliest onset of the P‐wave in the 12 leads to the latest activation of the coronary sinus catheter during sinus rhythm.^6^ All patients had undergone wide circumferential pulmonary vein isolation (CPVI) using a three‐dimensional mapping system (CARTO 3; Biosense Webster, Inc.) and a 3.5‐mm open‐irrigated tipped catheter (Navistar ThermoCool; ThermoCool SF, ThermoCool STSF, Biosense Webster, Inc.). The endpoint of pulmonary vein isolation (PVI) was defined as a bidirectional block between the left atrium and inside the CPVI area. Before January 2013, superior vena cava (SVC) isolation was performed if SVC‐triggered AF or rapid SVC firing was observed during the procedure. After January 2013, empiric SVC isolation was performed, except in patients without electrical potentials 10 mm above the right atrium‐SVC junction.^7^ A 10‐μg bolus of isoproterenol was administered at least 10 min after successful CPVI and atrial burst pacing up to a basic cycle length of 180 ms was conducted. We attempted to eliminate non‐pulmonary vein foci (non‐PV foci) and any ATA induced by programmed electrical stimuli with isoproterenol infusion. Non‐PV foci were defined as premature atrial contractions (PACs) from non‐PVs that initiated AF, whereas non‐PV PACs were defined as ectopic beats from non‐PVs that did not initiate AF.^8^ The origins of non‐PV foci and non‐PV PACs were defined as successful radiofrequency application sites. The absence of dormant conduction with adenosine‐triphosphate infusion was also confirmed. All patients were followed‐up at the outpatient clinic at 1, 3, 6, 9, and 12 months after ablation, and then every 6 months thereafter. The recurrence of AF was evaluated by electrocardiographic recordings or 24‐h ambulatory monitoring performed at 3, 6, 9, and 12 months after ablation, and then every 6 months thereafter. Patients with palpitations were encouraged to use portable electrocardiographic monitors (HCG‐801R; Omron). A second or third AF ablation session was attempted for ATA recurrence based on the discretion of the physicians.

### 2.4 Outcome measures

First, we compared the patient characteristics and procedural characteristics of the TBS and non‐TBS groups. Second, the ATA recurrence rates after a single/final ablation procedure without any AADs were examined. Recurrence was defined as ATAs lasting more than 30 s documented by ECG, 24‐h ambulatory monitoring, or portable electrocardiographic monitoring after a 2‐month blanking period after the ablation procedure without any AADs.^5^ Third, predictors of ATA recurrence after AF ablation in the TBS group and in the entire study population were studied. Fourth, the PMI avoidance rate and the period from the final ablation procedure to PMI in the TBS group were assessed. Clinical outcomes in patients with ATA recurrence after AF ablation were examined in detail. Finally, the heart rate before and after the ablation procedure were compared between groups. Heart rate before the ablation procedure was defined as the sinus rate on 12‐lead ECG on admission without AADs. The heart rate after the first ablation procedure was defined as the sinus rate without AADs at the outpatient clinic 1 month after discharge.

### 2.5 Statistical analysis

Categorical variables were expressed as numbers and proportions and compared using Fisher's exact test. Continuous variables were shown as mean and standard deviation or median with interquartile range and compared using the Student’s *t*‐test or Mann–Whitney *U* test. We performed a 1:2 propensity score‐matching analysis based on age, gender, AF type, and left atrial dimension to compare baseline and procedural characteristics of the TBS group with those of the non‐TBS group. The heart rate before and after the first ablation procedure were compared using a paired t‐test or a Wilcoxon signed‐rank test, as appropriate. The time‐to‐arrhythmia recurrence was estimated using the Kaplan–Meier method and compared using the log‐rank test. Cox‐proportional hazard regression models were used to examine factors associated with ATA recurrence after the first ablation procedure. Age, gender, and other baseline variables with *p* < .05 according to the univariate analysis were included as covariates in the multivariate analysis. Two‐sided *p* < .05 was considered statistically significant. JMP Pro 13® software (SAS Institute Inc.) was used for analysis.

## 3 RESULTS

### 3.1 Comparison of patient and procedural characteristics

The TBS group comprised 53 patients (mean age, 67.1 ± 7.0 years; males, 62.3%). All 53 patients in the TBS group underwent RFCA, although PMI was also indicated according to the guidelines in Japan.^9^ Twenty‐nine of 53 patients showed sinus pause for more than 3 s, followed by AF termination, resulting in bradycardia‐related symptoms only with the use of AADs, which meant that the PMI indication for these 29 patients was class IIa. The PMI indication was class I for the remaining 24 patients. The AF type was paroxysmal AF for all study patients. Patient characteristics and procedural summaries are shown in Table 1. No significant differences in age, gender, AF type, comorbidities, and medications were observed in the TBS and non‐TBS groups. There was a significant difference in the number of total ablation sessions of the groups. Although echocardiographic parameters, including left atrial dimension and left atrial volume index, did not significantly differ between groups, the PWD and IACT, which are indicators of atrial remodeling, were significantly longer in the TBS group. CPVI was successfully achieved in all study patients. Electrical isolation of the SVC was performed in 66.0% of the TBS group and in 81.1% of the non‐TBS group (*p* = .048). The incidence of non‐PV/SVC PACs during the first session was 30.2% in the TBS group and 9.4% in the non‐TBS group; this was statistically significant (*p* = .001). There were no significant differences in the PV reconnection rate, the incidence of non‐PV/SVC foci and the incidence of non‐PV/SVC PACs during the second session. The origins of non‐PV/SVC foci and non‐PV/SVC PACs are shown in Table 2. The origins of some non‐PV foci and non‐PV/SVC PACs were not determined because of failure to eliminate ectopic beats because of multiple origins or low reproducibility. Table 3 shows the bradycardia‐related characteristics of the TBS group. The maximum sinus pause was 5.3 ± 2.5 seconds. A history of syncope was observed for 18.9%; however, this rate did not significantly differ between groups with and without ATA recurrence. The prevalence of sinus pause (>3 s) with AADs was significantly higher in the group with ATA recurrence.

**TABLE 1:** Baseline patients' characteristics and procedural summary in patients with tachycardia‐bradycardia syndrome (TBS group) compared with patients without tachycardia‐bradycardia syndrome (non‐TBS group)

	Total (*N* = 159)	TBS group (*n* = 53)	Non‐TBS group (*n* = 106)	*p*‐value
Age, years	67.1 ± 7.0	67.1 ± 7.0	67.2 ± 7.0	.930
Gender, male	99 (62)	33 (62)	66 (62)	1.000
Body mass index	23.6 ± 3.0	23.6 ± 2.9	23.6 ± 3.5	.955
History of atrial fibrillation, months	34 (8.0–85.0)	35.5 (7.8–85.0)	27.5(10.0–102.0)	.574
Paroxysmal atrial fibrillation	159 (100)	53 (100)	106 (100)	1.000
CHADs score	1.1 ± 1.1	1.1 ± 1.0	1.1 ± 1.1	.696
CHA_2_Ds_2_‐Vasc score	2.3 ± 1.5	2.2 ± 1.3	2.3 ± 1.6	.563
Structural heart disease				
Ischemic heart disease	6 (32)	1 (17)	5 (39)	.664
Hypertrophic cardiomyopathy	7 (37)	2 (33)	5 (39)	.785
Post mitral valve replacement	2 (11)	1 (17)	1 (8)	.615
Post aortic valve replacement	1 (5)	1 (17)	0 (0)	.333
Post ventricular septal defect closure	1 (5)	1 (17)	0 (0)	.333
Dilated cardiomyopathy	1 (5)	0 (0)	1 (8)	.478
Cardiac sarcoidosis	1 (5)	0 (0)	1 (8)	.478
Hypertension	78 (49)	29 (55)	49 (46)	.320
Diabetes mellitus	24 (15)	7 (13)	17 (16)	.815
Congestive heart failure	10 (6)	2 (4)	8 (8)	.498
Prior stroke	20 (13)	5 (9)	15 (14)	.458
Medications				
Beta blocker	64 (40)	19 (36)	45 (43)	.494
Calcium channel blocker	4 (3)	1 (2)	3 (3)	.720
Sodium channel blocker	82 (52)	24 (25)	58 (55)	.313
Estimated glomerular filtration rate, ml/min/1.73m^2^	64.0 (54.9–73.8)	58.2 (51.7–68.0)	67.0 (58.5–76.4)	.009
Echocardiographic parameters				
Left atrial dimension, mm	37.6 ± 6.1	37.2 ± 5.5	37.8 ± 6.4	.563
Left atrial volume, ml	60.3 ± 19.1	58.7 ± 16.1	61.0 ± 20.4	.484
Left atrial volume index, ml/m^2^	36.1 ± 11.4	34.9 ± 11.3	36.6 ± 11.5	.376
Left ventricular end‐diastolic diameter, mm	46.7 ± 5.1	46.5 ± 4.4	46.7 ± 5.5	.772
Left ventricular end‐systolic diameter, mm	32.3 ± 5.5	32.0 ± 4.1	32.5 ± 6.0	.640
Left ventricular ejection fraction, %	55.8 ± 6.6	54.6 ± 6.7	56.4 ± 6.5	.102
P‐wave duration, ms	101.6 ± 16.2	109.3 ± 13.6	97.8 ± 16.1	<.001
Total session				
1	109 (69)	27 (51)	82 (77)	
2	44 (28)	25 (47)	19 (18)	.001
3	6 (4)	1 (2)	5 (5)	
Procedural characteristics in the 1st session				
CPVI	159 (100)	53 (100)	106 (100)	1.000
SVCI	121 (76)	35 (66)	86 (81)	.048
CTI ablation	43 (27)	13 (25)	30 (28)	.706
Procedural time, min	163.4 ± 56.0	173.1 ± 52.4	158.7 ± 57.3	.128
Fluoroscopic time, min	15.3 ± 11.5	16.9 ± 12.6	14.5 ± 10.9	.223
Non‐PV/SVC foci	4 (3)	2 (4)	2 (2)	.601
Elimination of non‐PV/SVC foci	2/4 (50)	1/2 (50)	1/2(50)	1.000
Non‐PV/SVC PACs	26 (16)	16 (30)	10 (9)	.001
Elimination of non‐PV/SVC PACs	8/26 (31)	5/16(31)	3/10(30)	.946
Dormant conduction of PVs	62 (39)	22 (42)	40 (38)	.731
Dormant conduction of SVC	17/121 (14)	9/35 (26)	8/86 (9)	.040
Intra‐atrial conduction time	109.0 ± 17.0	121.6 ± 10.3	105.1 ± 17.0	.006
Procedural characteristics in the 2nd session				
PV‐LA reconnection	43/51 (84)	21/26 (81)	22/25 (88)	.684
SVC‐RA reconnection	36/51 (71)	17/26 (65)	19/25 (76)	.754
CTI line reconnection	12/51 (24)	5/26 (19)	7/25 (28)	.742
Non‐PV/SVC foci	10/51 (20)	5/26 (19)	5/25 (20)	1.000
Elimination of non‐PV/SVC foci	7 /10 (70)	4/5 (80)	3/5 (60)	.490
Non‐PV/SVC PACs	5/51 (10)	3/26 (12)	2/25 (8)	.637
Elimination of non‐PV/SVC PACs	2/5 (40)	1/3 (33)	1/2 (50)	.709

*Note*: Values are presented as mean ± SD or as *n* (%) or median (interquartile range) as appropriate.

Abbreviations: CPVI, circumferential pulmonary vein isolation; CTI, cavo‐tricuspid itshumus; LA, left atrium; PV, pulmonary vein; RA, right atrium; SVCI, superior vena cava isolation.

**TABLE 2:** Origins of non‐PV/SVC foci and non‐PV/SVC PACs in the first and second ablation procedure

	TBS group (*n* = 53)	Non‐TBS group (*n* = 106)
Non‐PV/SVC foci in the first session		
Crista terminalis	0 (0)	1 (1)
PLSVC	1 (2)	0 (0)
Unknown	1 (2)	1 (1)
Non‐PV/SVC PACs in the first session		
Coronary sinus ostium	1 (2)	1 (1)
Crista terminalis	1 (2)	1 (1)
Posterior wall of the left atrium	1 (2)	0 (0)
PLSVC	2 (4)	0 (0)
Tricuspid valve annulus	0 (0)	1 (2)
Unknown	11 (21)	7 (13)
Non‐PV/SVC foci in the second session		
Coronary sinus ostium	1/25 (4)	1/25 (4)
Crista terminalis	2/25 (8)	2/25 (8)
Right atrium septum	1/25 (4)	0 /25 (0)
Unknown	1/25 (4)	2/25 (8)
Non‐PV/SVC PACs in the second session		
Coronary sinus ostium	1/25 (4)	0/25 (0)
Crista terminalis	0/25 (0)	1/25 (4)
Unknown	2/25 (8)	1/25 (4)

PAC, premature atrial contraction; PLSVC, persistent left superior vena cava; PV, pulmonary vein; SVC, superior vena cava.

**TABLE 3:** Bradycardia‐related characteristics in the TBS group

	Total (*N* = 53)	Recurrence (+) (*n* = 29)	Recurrence (−) (*n* = 24)	*p*‐value
Maximum sinus pause, sec	5.3 ± 2.5	5.4 ± 2.0	5.3 ± 3.2	.884
Sinus pause (>3 s) with AADs	29 (55)	21 (72)	8 (33)	.006
Syncope	10 (19)	5 (17)	5 (21)	.739

Abbreviation: AAD, anti‐arrhythmic drugs.

### 3.2 Arrhythmia recurrence and its predictors

**FIGURE 1:**
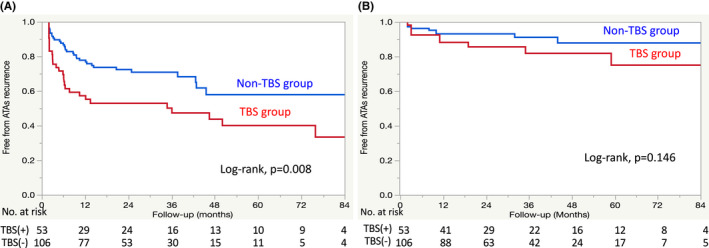
Kaplan–Meier survival analysis of freedom from atrial tachyarrhythmia recurrence without anti‐arrhythmic drugs (A) after the first ablation procedure and (B) after the final ablation procedure.

**FIGURE 2:**
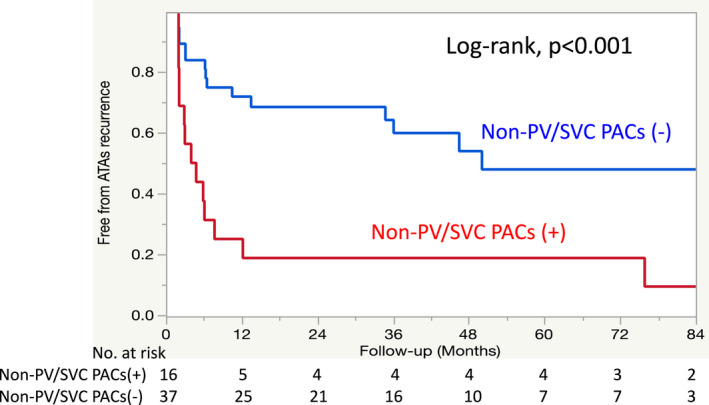
Kaplan–Meier survival analysis of freedom from atrial tachyarrhythmia recurrence in the TBS group.

A Kaplan–Meier survival analysis revealed that the ATA recurrence rate after a single ablation procedure without any AAD was significantly higher in the TBS group than in the non‐TBS group (51.0% vs. 38.5%; log‐rank *p* = .008) during a median follow‐up period of 37.7 months (interquartile range, 23.4–62.4 months) (Figure 1A). The ATA recurrence rates at 1 year after a single ablation procedure without AADs were 42.8% in the TBS group and 22.2% in the non‐TBS group. However, ATA recurrence after the final ablation procedure without AADs did not significantly differ between groups (log‐rank *p* = .146) (Figure 1B). The ATA recurrence rates at 1 year after the final ablation procedure without AADs were 86.1% in the TBS group and 93.1% in the non‐TBS group during a median follow‐up period of 26.7 months (interquartile range, 13.0–47.4 months). Table 4 shows the results of the multivariate analysis of ATA recurrence in the TBS group. The Cox‐proportional hazards regression model showed that the presence of non‐PV/SVC PACs was the only predictor of ATA recurrence after the first ablation procedure (odds ratio [OR], 3.38; 95% confidence interval [CI], 1.49–7.66; *p* = .004) (Figure 2). The results of the multivariate analysis of ATA recurrence in the entire study population, including the TBS and non‐TBS groups, are shown in Table S1. The predictors of ATA recurrence were TBS (OR, 2.28; 95% CI, 1.10–4.71; *p* = .028), the presence of non‐PV/SVC PACs (OR, 2.66; 95% CI, 1.15–5.84; *p* = .023), and history of congestive heart failure (OR, 3.89; 95% CI, 1.32–9.82; *p* = .016).

**TABLE 4:** Univariate and multivariate analysis for predictors of ATAs recurrence after the first ablation procedure in the TBS group

	Univariate analysis		Multivariate analysis	
	Odds ratio (95%CI)	*p*‐value	Odds ratio (95%CI)	*p*‐value
Gender, male	0.98 (0.47–2.16)	.968	1.30 (0.56–3.12)	.549
Age	0.98 (0.92–1.03)	.358	0.97 (0.91–1.04)	.379
Non‐PV/SVC PACs	3.33 (1.57–7.01)	.002	3.38 (1.49–7.66)	.004
Sinus pause (>3 sec) with AADs	2.58 (1.18–6.24)	.016	1.87 (0.82–4.66)	.153

Abbreviations: AADs, anti‐arrhythmic drugs; PAC, premature atrial contraction; PV, pulmonary vein; SVC, superior vena cava.

### 3.3 Avoidance of permanent PMI after AF ablation

**FIGURE 3:**
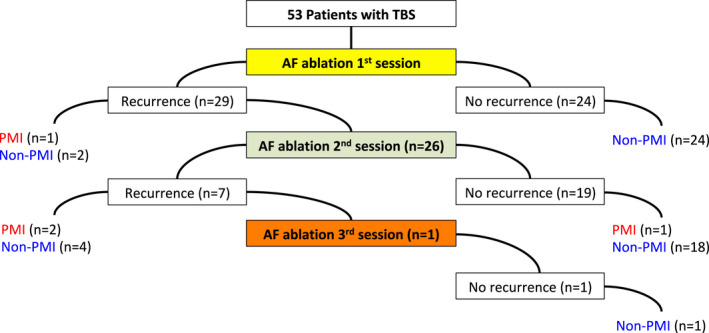
Clinical outcomes of patients with tachycardia‐bradycardia syndrome (TBS). The rate of permanent pacemaker implantation was 7.5% for patients with TBS. seven of 11 patients who experienced arrhythmia recurrence after the final ablation procedure avoided permanent pacemaker implantation.

A total of 53 patients with TBS underwent RFCA during the present study. Of 53 patients, only four underwent PMI. The median period from the final ablation procedure to PMI was 6.8 months. The PMI avoidance rate after the final ablation procedure for TBS and paroxysmal AF was 92.5% (Figure 3). Twenty‐nine of 53 patients had ATA recurrence after the first ablation procedure. Twenty‐six of those 29 patients underwent a second ablation session. One underwent the second session during the blanking period because AF recurrence was observed 1 week after the first ablation procedure and symptomatic sinus pause followed by AF termination occurred frequently. The patient chose a second ablation procedure rather than PMI. Twenty‐five patients underwent the second session after the blanking period. Among the remaining three patients who did not undergo the second session, two avoided PMI despite ATA recurrence and one underwent PMI 6 days after the initial ablation procedure because of symptomatic sinus pause followed by AF termination. Of the 26 patients who had undergone the second session, seven experienced ATA recurrence. Among those seven patients, two underwent PMI after the blanking period because of symptomatic sinus pause followed by AF termination. One underwent a third ablation procedure and did not experience ATA recurrence thereafter. The remaining 19 patients did not experience ATA recurrence after the second session. However, one of the 19 patients underwent PMI 14 days after the second session because of symptomatic sinus pause after short‐run PACs.

### 3.4 Clinical outcomes in patients with ATA recurrence after AF ablation

**FIGURE 4:**
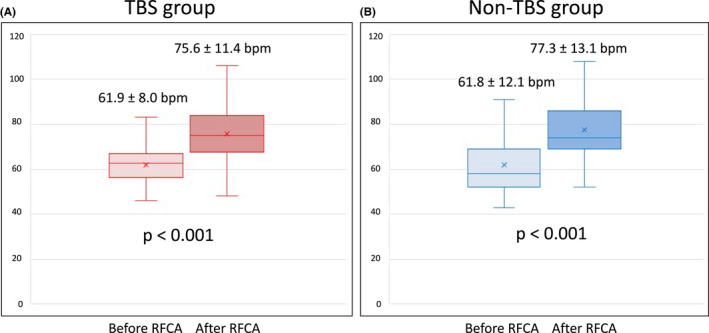
Heart rate before and after the first ablation procedure. Heart rates significantly increased after the first ablation procedure in both the TBS and non‐TBS groups.

Although 10 patients experienced ATA recurrence after the final ablation procedure, seven avoided PMI throughout the study period despite ATA recurrence. For three patients who experienced ATA recurrence after the first ablation procedure but did not undergo the second ablation session, ATA recurrence and sinus pause (>3 s) followed by tachycardia termination were detected using Holter ECG after the blanking period. However, this sinus pause followed by tachycardia termination lasted less than 4 s, which was shorter than that before RFCA, and was asymptomatic in all three patients. Additionally, ATA recurrence was not as frequent as that in those three patients and did not require PMI. For four patients who experienced ATA recurrence after the second ablation session, sinus pause (>3 s) followed by tachycardia termination or bradycardia‐related symptoms were not observed. AF persisted in only one of those four patients, and sinus rhythm maintenance was not attempted for that patient. Three of seven patients who experienced ATA recurrence after the final ablation procedure used AADs to maintain sinus rhythm after the blanking period of the final ablation procedure. Heart rates according to 12‐lead ECG before and after the ablation procedure were compared between groups. The TBS group had a significantly increased sinus rate after the ablation procedure (61.9 ± 8.0 bpm vs. 75.6 ± 11.4 bpm; *p* < .001) that was similar to that of the non‐TBS group (61.8 ± 12.1 bpm vs. 77.3 ± 13.1 bpm; *p* < .001) (Figure 4).

## 4 DISCUSSION

This study investigated the impact of RFCA on long‐term outcomes in patients with TBS and paroxysmal AF. One of the main findings of this study was that the ATA recurrence rate after the first ablation procedure was significantly higher for patients with TBS, which was associated with the presence of non‐PV/SVC PACs. However, the PMI rate after the final ablation procedure was much lower than the ATA recurrence rate after the first ablation session.

There are some possible mechanisms for this discrepancy. Atrial reverse remodeling after RFCA is one of the possible mechanisms. AF and sinus node dysfunction often coexist. AF is attributed to sinus node dysfunction and vice versa.^10^, ^11^ AF and atrial flutter cause atrial remodeling, which is characterized by the prolongation of the corrected sinus node recovery time and PWD and a decrease in heart rate. However, electrical atrial remodeling was shown to be reversible after termination of tachycardia.^12^, ^13^, ^14^ During the present study, patients with TBS exhibited prolonged PWD and IACT compared to patients without TBS, although the left atrial size and left atrial volume were similar between the groups. The PWD obtained using body surface ECG was a marker of atrial remodeling,^15^ and a prolonged PWD was significantly associated with AF recurrence after RFCA.^16^ Prolonged IACT also reflected atrial remodeling and was associated with new‐onset AF.^6^, ^17^ Therefore, prolonged PWD and IACT might have contributed to the higher ATA recurrence rates of patients with TBS. During the present study, the presence of non‐PV/SVC PACs that did not initiate AF during the first ablation procedure was more common in patients with TBS, which was significantly associated with ATA recurrence after the first ablation procedure for TBS. However, the induction rate of non‐PV PACs during the second session was lower than that during the first session, which might have resulted from the atrial reverse remodeling with the first ablation procedure. More attentive follow‐up might be needed, especially for patients with non‐PV/SVC PACs during the first ablation procedure because most of these patients experienced ATA recurrence within 1 year after the first ablation procedure. Hocini et al. demonstrated reverse remodeling of the sinus node function after AF ablation, which was characterized by increased mean and maximum heart rates and decreased corrected sinus node recovery time.^18^ This study demonstrated that the sinus rate at 1 month after the procedure was significantly higher than the sinus rate before RFCA in the TBS and non‐TBS groups. Patients with TBS had an increased sinus rate after RFCA despite the existence of sinus node dysfunction. An increased sinus rate after ablation might result not only from atrial reverse remodeling but also from the modification of ganglionated plexi around the pulmonary vein antrum. Ganglionated plexi ablation during pulmonary vein isolation results in an increased sinus rate.^19^ Symptomatic sinus pause followed by tachycardia termination was not observed in some patients with ATA recurrence after the first ablation procedure; therefore, repeated RFCA procedures, rather than PMI, might be preferred for those patients. As a result, the recurrence rate after the final ablation procedure was similar in the TBS and non‐TBS groups, which might have resulted from atrial reverse remodeling with repeated ablation procedures.

Discontinuation of AADs was another possible mechanism of the discrepancy in this study. Twenty‐nine (55% of the TBS group) patients used AADs before the first ablation procedure; however, AADs and nodal blocker agents such as beta‐blockers or calcium channel blockers were discontinued for at least five lives before the ablation procedure. Such drugs remained discontinued after the ablation procedure for most patients. Only three of the 29 patients needed AADs to maintain sinus rhythm after the blanking period of the final ablation procedure. However, the amount of AADs required to maintain sinus rhythm after AF ablation was lower than that required before AF ablation. Additionally, symptomatic sinus pause for more than 3 s was not detected in these three patients. A previous study demonstrated that the postablation AF burden was decreased despite AF recurrence after AF ablation,^20^ which might result in the discontinuation of AADs or a decrease in the amount of AADs required to maintain sinus rhythm. Therefore, fewer AADs after AF ablation might explain the discrepancy between higher ATA recurrence rates after the first ablation procedure and lower incidence of PMI.

The present study showed some conflicting data compared to previous reports of RFCA for TBS. The predictor of ATA recurrence after RFCA in the TBS group was the presence of non‐PV/SVC PACs in this study. However, Nakamaru et al. reported that the presence of residual PACs during the procedure was not associated with increased ATA recurrence after ablation. One of the possible reasons for this discrepancy is that non‐PV PACs might lack the substrate to initiate and maintain AF around the site of origin of non‐PV PACs.^8^ The TBS group in this study had prolonged PWD and IACT, suggesting advanced atrial remodeling. Patients with TBS might have sufficient atrial substrate to initiate and maintain AF by non‐PV/SVC PACs. Therefore, the elimination of residual PACs could lead to lower ATA recurrence rates after RFCA for TBS and paroxysmal AF. Although the efficacy of balloon ablation for paroxysmal AF has been reported,^21^, ^22^ RFCA rather than balloon ablation might be preferred for patients with TBS to eliminate non‐PV/SVC PACs. However, it is challenging to eliminate all residual non‐PV/SVC PACs because of multiple origins and low reproducibility. An increased heart rate after AF ablation was not observed in a previous study focused on TBS,^23^ which was in contrast to the results of this study.

Procedural differences might be another possible reason for the discrepancies. Segmental PVI was performed in study by Miyanaga et al.; however, we performed CPVI with an interlesion distance of less than 5 mm. The degree of ganglionated plexi modification might be different between segmental PVI and CPVI. Hayashi et al. reported that ATA recurrence rate after the final ablation procedure was significantly higher for patients with sick sinus syndrome; however, the present study did not significantly differ with regard to the ATA recurrence rate after the final procedure. They also demonstrated that the presence of non‐PV foci was one of the predictors of ATA recurrence after ablation.^24^ The aforementioned conflicting data might be attributed to differences in patient backgrounds or ablation protocols. In our study, the prevalence of patients who underwent SVC isolation was much higher than that reported by Hayashi et al.^24^ The SVC was one of the most common non‐PV foci of AF,^25^ and empiric SVC isolation in addition to CPVI was associated with better outcomes.^7^

The definition of the blanking period was associated with the recurrence rate after AF ablation and the discrepancy between the ATA recurrence rate and the PMI rate. A 3‐month blanking period is commonly used after AF ablation. However, many recent studies showed an association between early recurrence within 3 months of the blanking period and late recurrence. Therefore, the need to redefine the blanking period has been recently advocated.^26^, ^27^, ^28^, ^29^ Especially for cases of TBS, it is important to avoid underestimating the ATA recurrence after the ablation procedure because it is vital to determining whether PMI is necessary for patients with ATA recurrence after ablation. Four patients in the TBS group underwent a second ablation session between 2 and 3 months after the first ablation session. If we had used the 3‐month blanking period, then the recurrence rate would have been underestimated. Therefore, we used the 2‐month blanking period.

### 4.1 Study limitations

There were several limitations to this study that should be considered. First, this was a single‐center, retrospective cohort study. Therefore, selection bias might have occurred. Second, the study population of patients diagnosed with TBS and paroxysmal AF prior to RFCA was relatively small. The diagnosis of TBS was based on the Holter ECG or monitor ECG results. We did not routinely perform sinus node function evaluations including measurements of the sinus node recovery time. Therefore, the detection of TBS might be underestimated. For the alternative assessment of sinus node function, PWD and IACT might be useful because a previous study reported that those indicators were associated with sinus node dysfunction, including sinus node recovery time.^30^ Further prospective studies with a larger population are warranted to confirm the results of this study. Third, 29 patients diagnosed with TBS had sinus pause followed by tachycardia terminated only with the use of AADs. In most cases, AADs and nodal blocker agents remained discontinued after the ablation procedure. The use of fewer or more infrequent AADs and nodal blocker agents could have affected the PMI avoidance rate. However, drug‐induced TBS was a class IIa indication for PMI if AADs were necessary to control symptoms. Therefore, all 53 patients, including 29 patients with drug‐induced TBS, had indications for PMI according to the guidelines in Japan. Finally, the procedural protocol for SVC isolation differed before and after January 2013. However, only two patients in the TBS group and four patients in the non‐TBS group underwent AF ablation before January 2013. Therefore, a significant impact on the results of this study might not have been observed. To overcome these limitations, further prospective studies with larger populations are warranted to confirm the results of this study.

## 5 CONCLUSION

Patients with TBS had higher ATA recurrence rates after the first ablation procedure compared to those without TBS. The presence of non‐PV/SVC foci during the first ablation procedure was significantly associated with ATA recurrence in patients with TBS. In contrast to the ATA recurrence rate after the first ablation procedure, the PMI rate was much lower. Therefore, ATA recurrence after AF ablation did not necessarily result in PMI for patients with TBS.

## CONFLICT OF INTEREST

None.

## DECLARATIONS

*Approval of the research protocol*: The Institutional Review Board and Ethics Committee of the Tokyo Women's Medical University approved the study protocol (date/number: October 3, 2019/4190‐R).

## Supporting information


Table S1
