# Supplementary material for: Impact of atrial fibrillation ablation on long‐term outcomes in patients with tachycardia‐bradycardia syndrome
Source: J Arrhythm. 2022 May 19;38(4):598–607. doi: 10.1002/joa3.12738 (PMC9347207; doi:10.1002/joa3.12738)
Supplement: Supplementary file 1 — Table S1 [file JOA3-38-598-s001.docx]

| **Supplemental table 1.**  Univariate and multivariate analysis for predictors of ATAs recurrence in the entire study population | | | | | | | |  |
| --- | --- | --- | --- | --- | --- | --- | --- | --- |
|  |  | Univariate analysis | |  | Multivariate analysis | |  | |
|  |  | Odds ratio (95%CI) | p-value |  | Odds ratio (95%CI) | p-value |  | |
| **Sex, male** |  | 1.02 (0.62 – 1.74) | 0.945 |  | 0.79 (0.38 – 1.70) | 0.553 |  | |
| **Age** |  | 0.97 (0.93 – 1.00) | 0.053 |  | 0.98 (0.93 – 1.04) | 0.556 |  | |
| **History of atrial fibrillation, months** |  | 1.00 (1.00 – 1.01) | 0.004 |  | 1.00 (0.99 – 1.01) | 0.133 |  | |
| **Congestive heart failure** |  | 2.93 (1.12 – 6.34) | 0.031 |  | 3.89 (1.32 – 9.82) | 0.016 |  | |
| **TBS** |  | 1.95 (1.17 – 3.23) | 0.010 |  | 2.28 (1.10 – 4.71) | 0.028 |  | |
| **Non-PV/SVC PACs** |  | 3.19 (1.78 – 5.46) | <0.001 |  | 2.66 (1.15 – 5.84) | 0.023 |  | |
| **Dormant conduction of SVC** |  | 2.45 (1.13 – 4.87) | 0.024 |  | 1.07 (0.42 – 2.52) | 0.889 |  | |

PAC: premature atrial contraction, SVC: superior vena cava, TBS: tachycardia-bradycardia syndrome
